# Atypical Integration of Sensory-to-Transmodal Functional Systems Mediates Symptom Severity in Autism

**DOI:** 10.3389/fpsyt.2021.699813

**Published:** 2021-08-20

**Authors:** Shinwon Park, Koen V. Haak, Han Byul Cho, Sofie L. Valk, Richard A. I. Bethlehem, Michael P. Milham, Boris C. Bernhardt, Adriana Di Martino, Seok-Jun Hong

**Affiliations:** ^1^Institute for Basic Science, Center for Neuroscience Imaging Research, Sungkyunkwan University, Suwon, South Korea; ^2^Department of Biomedical Engineering, Sungkyunkwan University, Suwon, South Korea; ^3^Donders Institute of Brain, Cognition, and Behaviour, Radboud University Medical Center, Nijmegen, Netherlands; ^4^Otto Hahn Group Cognitive Neurogenetics, Max Planck Institute for Human Cognitive and Brain Sciences, Leipzig, Germany; ^5^Institute of Neuroscience and Medicine (INM-7), Forschungszentrum Jülich, Jülich, Germany; ^6^Department of Psychiatry, Autism Research Centre, University of Cambridge, Cambridge, United Kingdom; ^7^Brain Mapping Unit, Department of Psychiatry, University of Cambridge, Cambridge, United Kingdom; ^8^Center for the Developing Brain, Child Mind Institute, New York, NY, United States; ^9^Center for Biomedical Imaging and Neuromodulation, Nathan Kline Institute, New York, NY, United States; ^10^McConnell Brain Imaging Centre, Montreal Neurological Institute and Hospital, McGill University, Montreal, QC, Canada; ^11^Autism Center, Child Mind Institute, New York, NY, United States

**Keywords:** autism spectrum disoder, cortical hierarchy, low-level sensory, high-order system, connectopic mapping, subcortico-cortical connectivity

## Abstract

A notable characteristic of autism spectrum disorder (ASD) is co-occurring deficits in low-level sensory processing and high-order social interaction. While there is evidence indicating detrimental cascading effects of sensory anomalies on the high-order cognitive functions in ASD, the exact pathological mechanism underlying their atypical functional interaction across the cortical hierarchy has not been systematically investigated. To address this gap, here we assessed the functional organisation of sensory and motor areas in ASD, and their relationship with subcortical and high-order trandmodal systems. In a resting-state fMRI data of 107 ASD and 113 neurotypical individuals, we applied advanced connectopic mapping to probe functional organization of primary sensory/motor areas, together with targeted seed-based intrinsic functional connectivity (iFC) analyses. In ASD, the connectopic mapping revealed topological anomalies (i.e., excessively more segregated iFC) in the motor and visual areas, the former of which patterns showed association with the symptom severity of restricted and repetitive behaviors. Moreover, the seed-based analysis found diverging patterns of ASD-related connectopathies: decreased iFCs within the sensory/motor areas but increased iFCs between sensory and subcortical structures. While decreased iFCs were also found within the higher-order functional systems, the overall proportion of this anomaly tends to increase along the level of cortical hierarchy, suggesting more dysconnectivity in the higher-order functional networks. Finally, we demonstrated that the association between low-level sensory/motor iFCs and clinical symptoms in ASD was mediated by the high-order transmodal systems, suggesting pathogenic functional interactions along the cortical hierarchy. Findings were largely replicated in the independent dataset. These results highlight that atypical integration of sensory-to-high-order systems contributes to the complex ASD symptomatology.

## Introduction

The major symptoms defining autism spectrum disorder (ASD) include persistent deficits in social communication and interaction, and restricted/repetitive patterns of behaviors and interests, all of which either fully or at least partly involve high-order transmodal cortical regions (1–11). Because of such relevance for various phenotypes, high-order cognitive systems have long been a focus of ASD research in the past decades, providing a basis to construct seminal theories centered on atypical theory of mind (12, 13), or executive dysfunction (14–16). Given their importance, however, there are also earlier phenotypic features that can help with a diagnosis of ASD – i.e., low-level sensory or perceptual anomalies (17–24). These symptoms have been documented across all sensory modalities e.g., vision (25), auditory (26), touch (27), taste (28, 29), and smell (30, 31), affecting up to 95% of children with ASD as a form of either hypo- or hyper-sensitivity or both (32, 33). Indeed, the most up-to-date edition of the Diagnostic and Statistical Manual of Mental Disorders (DSM-5) includes these sensory symptoms as a part of autism phenotypes (34), suggesting their clinical importance to characterize this pervasive condition.

Accumulating evidence indicates that atypical development of early primary regions is not limited to sensory-related problems, but may cascade into the development of higher-order transmodal functional systems (35, 36). Low-level sensory areas such as visual and auditory cortices have a critical period during their brain development (37–39), for which abnormal synaptic connections or neurotransmitter system dysfunction may affect the formation of a large-scale cortical hierarchy (40–42). Previous neuroimaging studies in ASD provide supportive findings, with a demonstration of atypical functional changes in multiple sensorimotor and subcortical areas as well as in their connections toward the high-order brain networks in infants at high risk for ASD (43–46). In parallel, several studies also demonstrated that aberrant responses to sensory stimuli reliably predict symptom severity at a later stage in ASD (19, 20, 47). These results, together with perception-related theoretical accounts for ASD such as predictive coding (48) or weak central coherence (49), highlight the potential mechanisms involving atypical functional interplay between the low-level sensory (bottom-up sensory signal transmission) and higher-order transmodal (top-down modulation) systems in ASD. Despite the significance of this model, how the interaction between these hierarchically distinct two subsystems contribute to each behavioral symptom of ASD remains poorly understood, mainly because of lack of analytical tools to integrate multiple brain systems and behaviors in a mechanistically meaningful way.

Over the last two decades, resting-state functional magnetic resonance imaging (rs-fMRI) has been instrumental to elucidate large-scale canonical networks of intrinsic functional connectivity and their relationship to complex human behaviors (50, 51). Providing testable measurements to probe the macroscale functional organization without requiring explicit tasks during the scan, rs-fMRI became one of the most efficient tools across multiple research domains, especially for clinical neuroscience which has been actively seeking the reliable biomarkers (52). Beyond conventional approaches focusing on connectivity profiles of specific regions, recent methods increasingly characterize distributed functional networks (53–55). A recent study of our group utilizing a dimensionality reduction technique on rs-fMRI data revealed cortex-wide gradients that describe a continuous transition of connectivity profiles along the multiple cortical hierarchy axes including sensory-to-transmodal and visual-to-sensorimotor streams (55). Notably, mapping such topological organization is not confined to the whole brain space but has been also applied to pre-specified cortical regions of interest corresponding to domain specific processes. Recapitulating a biologically meaningful functional organization of visual (54), somatosensory (54, 56), thalamus (57), hippocampus (58, 59), and insula (60, 61) areas, this approach, called “connectopic mapping,” becomes a versatile tool to probe the details of local circuit systems in terms of their intrinsic functional connectivity organization.

Here, we combined connectopic mapping and seed-based analyses to map biological substrates underlying the co-occurring deficits of low-level sensory/motor and high-order behavioral symptoms in ASD. The current study is grounded on the premise that normative sensory development will provide the foundational building blocks for the formation of higher-order cortical systems in the later developmental stages. Based on this notion, we focused on four major sensory/motor systems, namely, sensory (S1), motor (M1), visual (V1) and auditory (A1) cortices, and investigated their relationship to high order transmodal systems as well as subcortical structures. Our framework was 3-fold: (i) We first established the functional organization of each targeted sensory/motor area in neurotypical (NT) and ASD groups by comparing their connectopic gradient maps. In ASD, we further, examined the association of each gradient with key behavior symptoms. (ii) After assessing the functional topology of each sensory/motor area, we compared the detailed seed-based functional connectivity profiles of the sensory/motor and subcortical areas between ASD and NT. Likewise, functional connectivity within high-order networks was also investigated to delineate a full picture of functional relationships between the systems within the cortical hierarchy. (iii) Finally, based on these results, we conducted a series of integrative mediation analyses to examine the impact of sensory/motor networks on high-order organization and consequently, their collective role in the manifestation of behavioral symptoms in ASD. We hypothesized that (i) the ASD group will show atypical functional gradient profiles across multiple sensory/motor areas, (ii) given recent studies demonstrating disrupted cortical hierarchies in autism (50), their high-order transmodal systems and relationship with low-level sensory/motor networks will also reveal atypical representations of connectivity, and (iii) altered functional integration between those subsystems may mediate the key behavioral symptoms in ASD.

## Materials and Methods

### Subjects

We extracted two waves of datasets from the Autism Brain Imaging Data Exchange repositories (ABIDE-I & II; http://fcon_1000.projects.nitrc.org/indi/abide) for the purpose of discovery and replication of findings. Inclusion criteria were similar to our previous studies (62–64). Briefly, the data acquisition sites were selected only when there were more than 10 individuals per group, including both children and adults with ASD as well as neurotypical (NT) controls. Due to the low prevalence of females in the ABIDE-I dataset, all analyses were restricted to males. After quality control, a total of 220 individuals were included in the final analyses set from three sites (107 ASD [age, mean ± SD = 20.9 ± 8.03], 113 age-matched NT [age = 19.3±7.31]): (i) NYU Langone Medical Center (NYU, 35/51 ASD/NT); (ii) University of Utah, School of Medicine (USM, 52/40 ASD/NT); (iii) University of Pittsburg, School of Medicine (PITT, 20/22 ASD/NT). For ABIDE-II, inclusion criteria were similar except that females were included because of their high rate of data availability (*n* = 19). A total of 116 individuals from 3 sites were included (57 ASD, 59 NT): (i) NYU Langone Medical Center (NYU, 29/19 ASD/NT); (ii) Institut Pasteur/Robert Debré Hospita (IP, 11/21 ASD/NT); (iii) Trinity Centre for Health Sciences, Trinity College Dublin (TCD, 17/19 ASD/NT). The ASD and NT groups in this dataset showed a difference in their age (ASD: mean ± SD = 12.1 ± 5.89, NT: mean ± SD = 16.5 ± 8.21; *t* = 3.3, *p* < 0.001). Therefore, in all replication analyses, we included both age and sex as nuisance covariables. Moreover, we assessed the reproducibility of our main findings while excluding female subjects from the replication dataset. Individuals with a diagnosis of Autistic, Asperger's, or Pervasive Developmental Disorder Not-Otherwise-Specified were all included in the ASD group. Diagnosis was established based on a structured or unstructured in-person interview by an expert clinician using the Autism Diagnostic Observation Schedule (ADOS) and/or the Autism Diagnostic Interview-Revised (ADI-R). Total scores along with those for reciprocal social interactions (ADOS-S), communication/language (ADOS-C), and restricted/repeated behaviors (ADOS-R) were provided in the ABIDE phenotypic dataset (65). Classic total ADOS scores were calculated by adding the reciprocal social interaction and communication/language subscores (66). Demographic and clinical characteristics for ABIDE-I (Table 1) and ABIDE-II (Supplementary Table 1), as well as participant profiles based on ADOS modules (Supplementary Table 2) are provided.

**Table 1:** Demographic and clinical characteristics of the ABIDE-I dataset.

	**ASD (*n* = 107)**	**NT (*n* = 113)**	**Statistical test**	
	**Mean (SD)**	**Mean (SD)**	***t***	***p***
Age (years)	20.9 (8.03)	19.3 (7.31)	1.48	0.14
Mean FD	0.11 (0.10)	0.10 (0.11)	0.40	0.69
ADOS-total	12.7 (3.68)	NA	NA	NA
ADOS-repetitive behavior	2.02 (1.50)	NA	NA	NA
ADOS-communication	4.25 (1.51)	NA	NA	NA
ADOS-social	8.40 (2.65)	NA	NA	NA

*ASD, autism spectrum disorder; NT, Neurotypical control; SD, standard deviation; FD, framewise displacement; ADOS, Autism Diagnostic Observation Schedule; NA, not available*.

### MRI Data Acquisition

Data was acquired on Siemens (NYU, PITT, USM) or Philips (IP, TCD) 3T MRI scanners across five different sites.

At *NYU*, Allegra with 3D-TurboFLASH was used for T1-weighted (T1w) data acquisition (matrix = 256 × 256; 1.3 × 1.0 × 1.3 mm^3^ voxels; TR = 2530 ms; TE = 3.25 ms; TI = 1100 ms; flip angle = 7°), and 2D-EPI was used for rs-fMRI (matrix = 80 × 80; 180 volumes, 3.0 × 3.0 × 4.0 mm^3^ voxels; TR = 2,000 ms; TE = 15 ms; flip angle = 90°). At *PITT*, Allegra with 3D-MPRAGE was used for T1w data acquisition (matrix = 269 × 269; 1.1 × 1.1 × 1.1 mm^3^ voxels; TR = 2100 ms; TE = 3.93 ms; TI = 1000 ms; flip angle = 7°) and 2D-EPI was used for rs-fMRI (200 volumes, 3.1 × 3.1 × 4.0 mm^3^ voxels; TR = 1500 ms; TE = 35 ms; flip angle = 70°; matrix = 64 × 64). At *USM*, T1w data were acquired on a TrioTim using 3D-MPRAGE (matrix = 240 × 256; 1.0 × 1.0 × 1.2 mm^3^ voxels; TR = 2300 ms; TE = 2.91 ms; TI = 900 ms; flip angle = 9°) and 2D-EPI for rs-fMRI (matrix = 64 × 64; 240 volumes; 3.4 × 3.4 × 3.0 mm^3^ voxels; TR = 2,000 ms; TE = 28 ms; flip angle = 90°). At *TCD*, Achieva with 3D-MPRAGE was used for T1w data acquisition (matrix = 256 × 256; 0.9 × 0.9 × 0.9 mm^3^ voxels; TR = 3000 ms; TE = 3.90 ms; TI = 1150 ms; flip angle = 8°) and 2D-EPI for rs-fMRI (matrix = 80 × 80; 210 volumes; 3.0 × 3.0 × 3.2 mm^3^ voxels; TR = 2,000 ms; TE = 27 ms; flip angle = 90°) at TCD. At *IP*, data were acquired on Achieva using 3D-MPRAGE for T1w (matrix = 240 × 240; 1 × 1 × 1 mm^3^ voxels; TR = 2500 ms; TE = 5.60 ms; flip angle = 30°) and 2D-EPI for rs-fMRI (matrix = 64 × 63; 85 volumes; 3.59 × 3.65 × 4 mm^3^ voxels; TR = 2700 ms; TE = 45 ms; flip angle = 90°).

### MRI Processing

#### Structural MRI

T1w images were processed with FreeSurfer (v5.1; http://surfer.nmr.mgh.harvard.edu/) to perform intensity normalization, skull stripping, registration to stereotaxic space, and tissue segmentation. White and pial surfaces are reconstructed by fitting a triangular surface tessellation and going through automated topology correction and surface deformation. These surfaces are inflated and then spherically registered to an atlas, *fsaverage*, with regards to the gyral and sulcal curvature.

#### rs-fMRI

rs-fMRI data already preprocessed by the Configurable Pipeline for the Analysis of Connectomes (C-PAC; https://fcp-indi.github.io/) were provided from the Preprocessed Connectomes initiative (http://preprocessed-connectomes-project.org/abide/). Briefly, preprocessing included slice-time correction, head motion correction, skull stripping, and intensity normalization. Effects of head motion, white matter and cerebrospinal fluid signals were statistically removed using CompCor (67). Following band-pass filtering (0.01–0.1 Hz), rs-fMRI data were registered to the MNI152 standard space using linear and non-linear transformations. After verification of surface alignment, individual rs-fMRI data were interpolated along the corresponding mid-thickness surfaces and resampled to the Conte69 template (https://github.com/Washington-University/Pipelines). Surface-based spatial smoothing was applied with a 5 mm full width at half maximum kernel.

### Connectopic Mapping of Low-Level Sensory/Motor Regions

#### Regions of Interest

**Figure 1:**
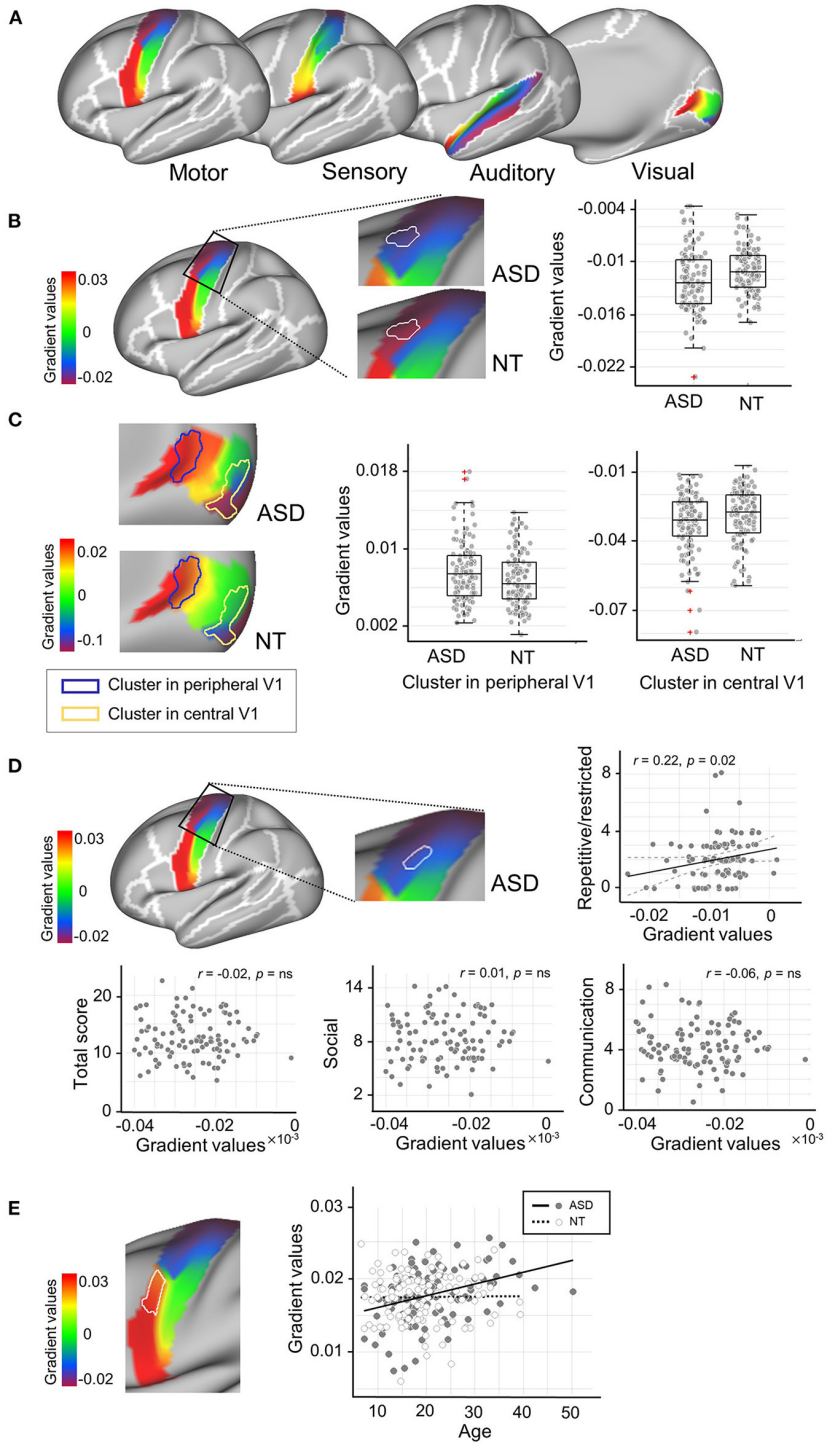
Connectopic mapping analysis. **(A)** The functional gradient maps of neurotypicals (NT) for low-order sensory/motor regions are presented. The motor (precentral), sensory (postcentral) and auditory (transverse and superior temporal) masks are derived from the Desikan-Killiany atlas, while the visual mask is from the Brodmann Area (BA) atlas corresponding to the primary visual area (BA 17). **(B)** The cluster that showed significant group difference in M1_Upper_ is marked in solid, white outlines. More extreme negative gradient scores were found in the ASD group, indicating an abnormally more segregated intrinsic functional connectivity (iFC). **(C)** The clusters that showed significant group differences in V1_Central_ and V1_Peripheral_ are marked in yellow and blue solid outlines, respectively. This central-to-peripheral gradient expansion in ASD indicates that iFC in the visual region becomes more segregated in ASD. **(D)** A cluster in M1_Upper_ showed significant associations with ADOS scores for restricted and repetitive behavior in ASD. **(E)** The cluster that showed a significant age-by-group interaction effect is marked in solid, white outlines. ADOS, Autism Diagnostic Observation Schedule.

Connectopic mapping targeted four primary sensory/motor areas (i.e., M1, S1, V1, A1). Three of them (i.e., M1, S1, A1) were derived from the Desikan-Killiany (DK) atlas (https://surfer.nmr.mgh.harvard.edu/fswiki/CorticalParcellation), where M1 and S1 correspond to the precentral and postcentral gyri, respectively, while A1 consisted of the transverse temporal and superior temporal gyri. V1 was delineated from a separate Brodmann Areas (BA) atlas, (https://surfer.nmr.mgh.harvard.edu/fswiki/BrodmannAreaMaps) to accurately capture the primary visual cortex corresponding to BA17 (Figure 1A).

#### Connectopic Mapping

The connectopic map represents how the vertex-wise connections of intrinsic functional connectivity (iFC) between the targeted sensory/motor area and the rest of neocortex vary topographically within the given ROI. The procedures to compute this map consist of two steps, a construction of a cortex-wise similarity matrix based on the functional connectivity profile followed by dimensionality reduction based on Laplacian Eigenmaps. Details can be found in Haak et al. (54). To reduce the computational cost during the calculation of similarity matrices, we first performed a lossless reduction of the functional time-series in the rest of the neocortex (outside the targeted sensory/motor region) using singular value decomposition (= # of time-series × # of eigenvectors). We then correlated this neocortical timeseries to those of the target sensory/motor area (= # of vertices at the given sensory/motor area × # of eigenvectors), which was in turn used to construct a similarity matrix with the η^2^ coefficient. This matrix represents the vertex-wise connectopic similarity between the sensory/motor area and the rest of the brain. For the second step, the algorithm for Laplacian eigenmaps was applied to the similarity matrices, in order to obtain a representation of the dominant modes of change in iFC across the ROIs. We used the generalized Procrustes analysis (https://brainspace.readthedocs.io/en/latest/) to align these resultant connectopic components of each individual (target) to a group-level gradient template (source) that was derived from an average connectivity matrix comprised of NT individuals.

### Seed-Based Functional Connectivity Networks

While the connectopic gradient approach effectively captures the regional topology of functional organization in a given system, it does not necessarily elucidate detailed pairwise connectome-level contribution to the macroscopic functional network. To achieve that, we stratified the whole brain into the low-level sensory/motor, subcortical and high-order transmodal systems, and assessed their interaction in the following systematic order: (i) within sensory/motor areas, (ii) between sensory/motor and subcortical areas, and (iii) within high-order transmodal areas. With these stratified brain systems, we performed a targeted seed-based connectivity analysis, in which individual iFC matrices were constructed by correlating the functional time-series across the predefined parcels. The resultant correlation values were r-to-z transformed. To obtain the boundaries of these parcels, we used the Yeo-Krienen 17 (Yeo-17) network atlas [**Figure 3C**; (68)].

#### Low-Level Functional Networks

**Figure 2:**
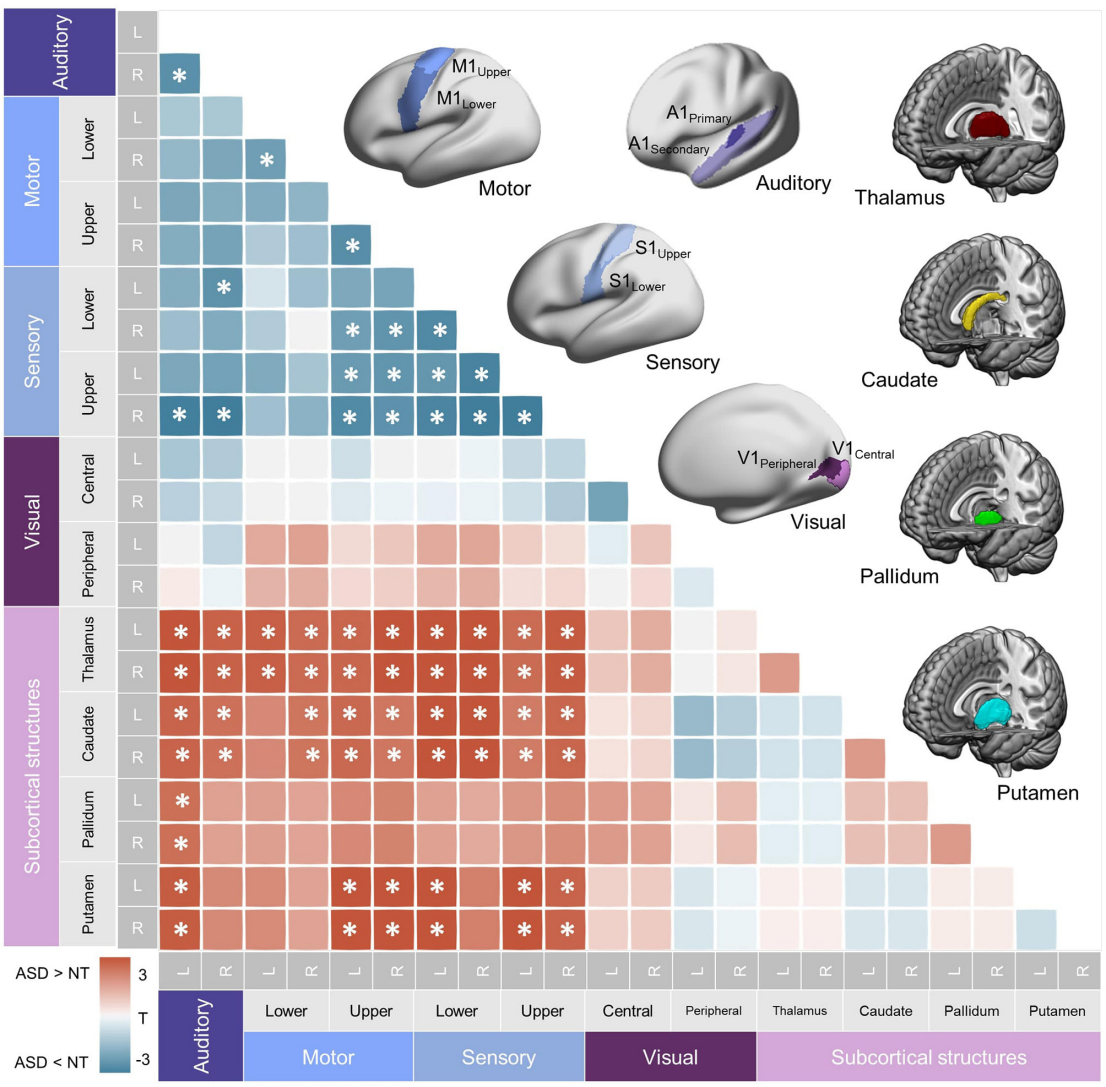
Subcortical-primary sensory/motor network. *Top right*. Parcellations used for the seed-based functional connectivity analysis of within-primary sensory/motor and subcortical networks are shown. (See **Materials and methods** for details.) *Bottom left*. A heatmap representing correlation coefficients of intrinsic functional connectivity (iFC) in subcortical-primary/motor networks. Significant group differences are shown with asterisks (FDR = 0.05). Hypoconnectivity (ASD < NT) was found in the within-primary sensory/motor network, and hyperconnectivity (ASD > NT) was found between subcortical structures and sensory/motor cortices, collectively suggesting an atypical diverging pattern of iFC in ASD. L, left; R, right; NT, Neurotypical controls; ASD, Autism Spectrum Disorder.

As the original Yeo-17 network atlas merged the auditory cortex into the somatomotor area without distinction, the boundary of the parcels for low-level areas had to be modified by intersecting them with the ROIs used for the connectopic mapping (i.e., DK & BA atlases; Figure 1A). Specifically, a total of 7 parcels for each hemisphere was included as primary sensory/motor networks. For the first 4 parcels (M1_Upper_, M1_Lower_, S1_Upper_, S1_Lower_), the somatomotor networks from the Yeo-17 atlas, which are originally subdivided into two networks (upper and lower) along the dorsoventral axis, were overlayed on the precentral (M1) and postcentral (S1) cortices corresponding to the DK atlas. This procedure yields parcels that closely reflect the functional topological organization (based on Yeo17 atlas) within the anatomically defined boundaries of M1 and S1 (from the Desikan-Killiany atlas). Similarly, the next 2 parcels (V1_Central_ and V1_Peripheral_) were delineated by overlaying the central and peripheral visual networks from the Yeo-17 atlas on the V1 cortex based on the BA atlas. The final parcel, auditory cortex (A1), was derived from the DK atlas including the transverse temporal and superior temporal gyri. Together with subcortical structures including the thalamus, caudate, pallidum and putamen, which parcels were derived from the Human Connectome Project parcellation (69, 70), this sensory/motor-subcortical network resulted in a 22 × 22 functional connectivity matrix (7 × 2 bilateral sensory/motor areas + 4 × 2 bilateral subcortical structures; Figure 2).

#### High-Order Functional Networks

**Figure 3:**
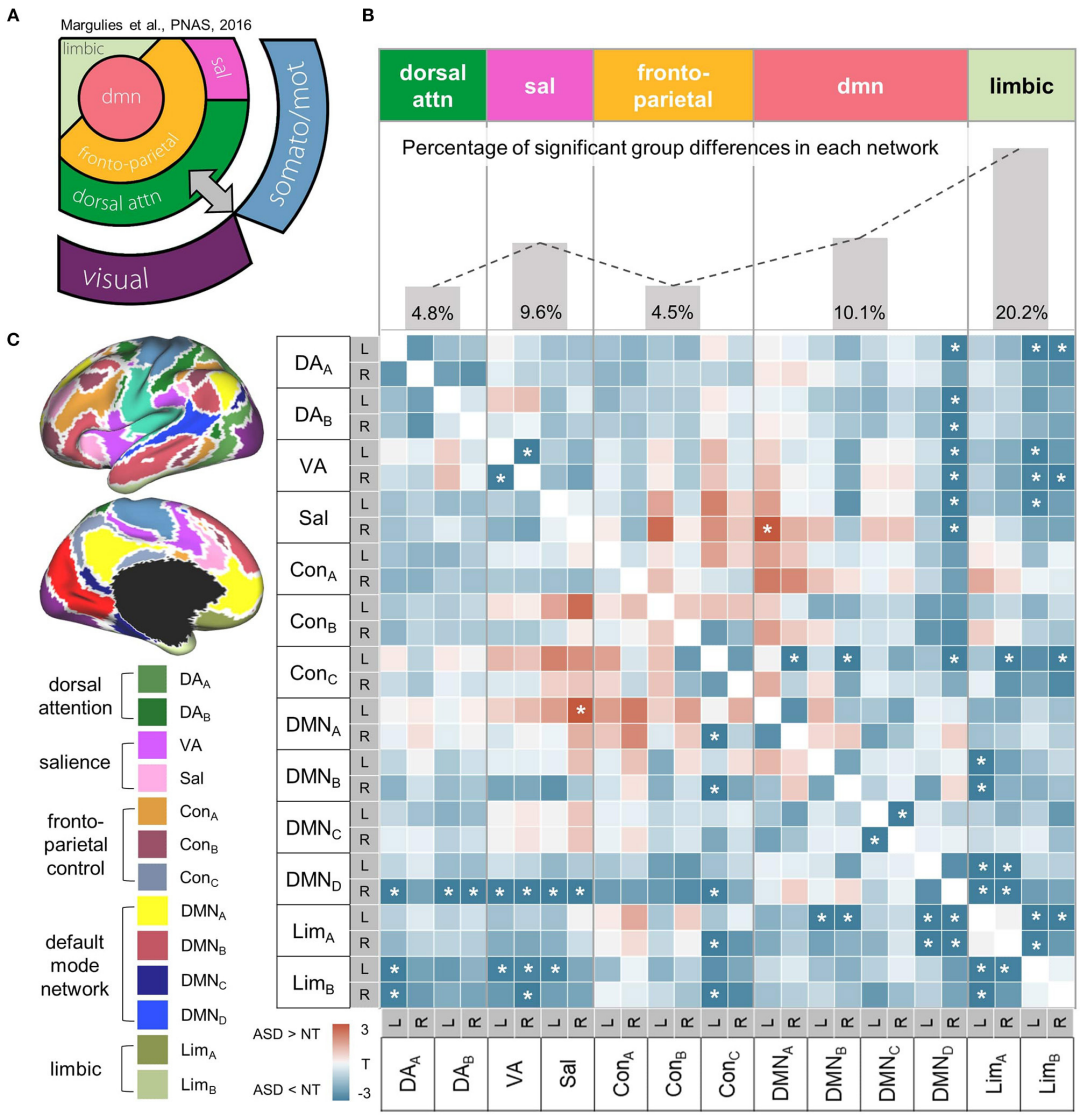
Within-high-order transmodal network. **(A)** A schematic diagram showing the hierarchical relationships of canonical resting-state networks. The figure component adapted from Margulies et al. (55). **(B)** *Top*. The proportion of altered connectivity in ASD shows an increasing trend along the hierarchy of high-order networks that was aligned based on the schematic diagram on the left. *Bottom*. A heatmap representing correlation coefficients of intrinsic functional connectivity (iFC) within high-order networks. Markedly decreased iFC in ASD suggests a less integrated high-order transmodal system. Significant group differences in iFC between ASD and NT are shown with asterisks (FDR = 0.05). **(C)** High-order network parcels were derived from the Yeo-Krienen 17 resting state networks (68). DA, dorsal attention network; VA, ventral attention network; Sal, salience network, Con, frontoparietal control network; DMN, default mode network; Lim, limbic network.

The parcels from the original Yeo-17 network atlas, except for the primary sensory/motor areas, were employed to construct the high-order iFC matrix. As a result, a 26 × 26 matrix was calculated including the following high-order networks: dorsal attention (DA_A_, DA_B_), ventral attention (VA), salience (Sal), frontoparietal control (Con_A_, Con_B_, Con_C_), default mode network (DMN_A_, DMN_B_, DMN_C_, DMN_D_), and limbic networks (Lim_A_, Lim_B_) (Figure 3C).

### Statistical Analysis

#### Connectopic Gradients

Functional connectopic maps for each sensory/motor region were statistically compared between ASD and NT using surface-based linear models implemented in a Matlab toolbox, SurfStat (http://www.math.mcgill.ca/keith/surfstat/). A series of analyses were also conducted to assess correlations with ADOS total and sub-scores in functional connectopic maps for each sensory/motor region. Finally, age-by-group interaction effects were tested to compare “cross-sectional” aging effects between ASD and NT. All models included age, acquisition site, and framewise displacement (and sex for the replication data) as covariates and were conducted as two-tailed tests. Correction for family-wise errors due to multiple comparisons were performed using the random field theory method with a cluster level correction of p_FWE_ < 0.05 (cluster defined at *p* = 0.025).

#### Functional Connectivity

Similar to the gradient analysis, individually identified functional connectivity matrices were also statistically compared between ASD and NT groups. We fitted a linear model to test for group effects while controlling for age, acquisition site, and framewise displacement as covariates. We also assessed the correlations between functional connectivity and the ADOS scores (i.e., total and subdomain scores). Finally, age-by-group interaction effects were tested for all regions that showed significant group differences of functional connectivity. The significance of these parcel-wise tests was corrected for multiple comparisons using a false discovery rate (FDR) procedure.

#### Mediation Analyses

The impact of low-level sensory/motor iFC on high-order connectivity networks and ASD-related symptoms was tested with a series of mediation models. In principle, testing for mediation is a two-step regression with three major components, i.e., a predictor (independent variable), a responder (dependent variable) and a mediator. First, the interaction of the predictor and responder is controlled for when testing the effects of the mediator to responder. A complete mediation occurs when the effect of the mediator is over and above the effect variance captured by predictor-responder. In our model, both predictor and mediator were taken from the results of the previous analyses. Specifically, the predictor was computed as an average of within-primary sensory/motor iFCs that showed the statistical associations with ADOS scores, while the mediator was calculated in the same manner using within-high-order transmodal iFCs. The responder was a severity score of each symptom in ASD (i.e., total, social interaction, communication, and restricted/repeated behaviours ADOS scores). Please note that the statistical relationship between the predictor and mediator is designated as effect “*a,”* and that between the mediator and responder as “*b,”* while between the predictor and responder as “*c.”* In our study, effect “*a”* tested whether sensory/motor iFC is correlated with high-order iFC, while effect “*b”* tested whether high-order iFC is related to ASD-related symptom severity, controlling for sensory/motor iFC. Finally, effect “*a* × *b*” tested the mediation effect by examining the significance of c - c' (c' refers to a direct effect after taking into account the mediation effect). A significant mediation effect suggests that high-order transmodal iFCs explain a significant proportion of the association between sensory/motor iFCs and ASD symptom severity. All coefficients were tested for significance using bootstrap tests (100,000 iterations) with the Mediation Toolbox for Matlab [https://github.com/canlab/CanlabCore (71, 72)].

### Results

#### Connectopic Profiling of Primary Somatosensory Regions

We compared the dimension-reduced, intrinsic functional connectivity (iFC) gradients of representative sensory/motor systems between ASD and neurotypical (NT) groups. Please note that in the Laplacian Eigenmaps, eigenvalues are sorted in ascending order and more variance is explained by the first eigenvalues (73). According to this criterion, we therefore, chose to focus on the first gradient at each sensory area, because they represent biologically meaningful functional organizations (e.g., retinotopic, somatotopic, and tonotopic maps), established by other independent modalities such as electroneurophysiology (74–77).

First, we profiled the NT group to establish normative functional gradients across the targeted primary sensory/motor systems (Figure 1A). Specifically, S1 and M1 showed a well-established somatotopic organization which follows a dorsomedial-to-ventrolateral gradient [i.e., homunculus; (74)] along both pre- and post-central gyri. V1 displayed an accurate representation of eccentricity i.e., a property representing the foveal-to-peripheral gradient in the receptive field (78), extending from posterior to anterior along the medial surface of the calcarine cortex. Despite less consensus on the tonotopic mapping of the auditory cortex (79), the gradient in A1 ran in anterior-to-posterior direction, including the transverse temporal gyrus (i.e., Heschl's gyrus) and planum temporale.

Gradient patterns were globally similar between the NT and ASD groups. However, vertex-wise statistical analyses revealed marked anomalies specific to ASD. In particular, the upper left motor cortex showed a significant change of more extreme negative gradient scores (i.e., abnormally more segregated iFC) in ASD (*t* = −2.28, p_FWE_ ≤ 0.05, Cohen's *d* = 0.4; Figure 1B). Alterations were also observed in the right visual cortex, showing both increased (V1_Peripheral_: t = 2.06, p_FWE_ ≤ 0.05, Cohen's *d* = 0.2) and decreased (V1_Central_: t = −1.97, p_FWE_ ≤ 0.05, Cohen's *d* = 0.3) gradients in the ASD group (Figure 1C). As similar to the M1 finding, this central-to-peripheral gradient expansion suggests that the iFC in ASD becomes excessively more segregated within the primary visual area. There were no significant group differences for sensory and auditory regions. These gradient changes in ASD were largely replicated in the ABIDE-II dataset, regardless of whether including both sexes or only males (Supplementary Figure 1).

When associating these gradient values with symptom severity, the M1 region (adjacent to the area we found significant group differences above) revealed a correlation to the ADOS scores for repetitive and restricted behaviors in ASD (*r* = 0.22, p_FWE_ = 0.04; Figure 1D) but not for the other two subdomain scores (i.e., social interaction and communication). Finally, a significant age-by-group interaction effect was found in M1 where the gradient values increased as aging in ASD, which pattern indicates a shift toward more extreme values (*F* = 6.14, *p* = 0.01; Figure 1E), similar to the results from the group difference analysis. These findings were not replicated in ABIDE-II.

#### Seed-Based Functional Connectivity Analysis

Following the gradient analyses, we sought to understand the relationship between low-level primary sensory/motor and the high-order transmodal systems in ASD by using the seed-based iFC approaches.

##### Within-Primary Sensory/Motor and Subcortical Networks

We first assessed how iFCs in these low-level sensory/motor related areas were affected in ASD. This analysis demonstrated hypoconnectivity between S1 and A1, S1 and M1_Upper_, and within the S1 and M1 areas (FDR = 0.05), corroborating the previous reports of dysconnectivity syndromes in ASD. Notably, however, assessing the sensory/motor-subcortical iFC showed rather marked hyperconnectivity across virtually all pairs of the structures in ASD, suggesting that iFCs in ASD are composed of mixed anomalies (increase and decrease), depending on the functional systems investigated (Figure 2A).

##### Within-High-Order Transmodal Systems

Beyond the analyses focusing on the low-level system, we further examined transmodal networks, independently from the sensory/motor iFC. The group comparison of iFC revealed markedly decreased connectivity across different functional networks in ASD, suggesting their less integrated transmodal systems. Notably, the proportion of such hypoconnectivity in ASD shows a trend of increase along the presumed order of cortical hierarchy (Figure 3A), for instance, from the dorsal attention networks showing mildly affected iFCs (percentage of significant t-score of hypoconnectivity = 4.8%) to the default mode (10.1%) and limbic (20.2%) systems with more severely decreased iFC patterns (Figure 3B).

##### Age-by-Group Interaction Effects

**Figure 4:**
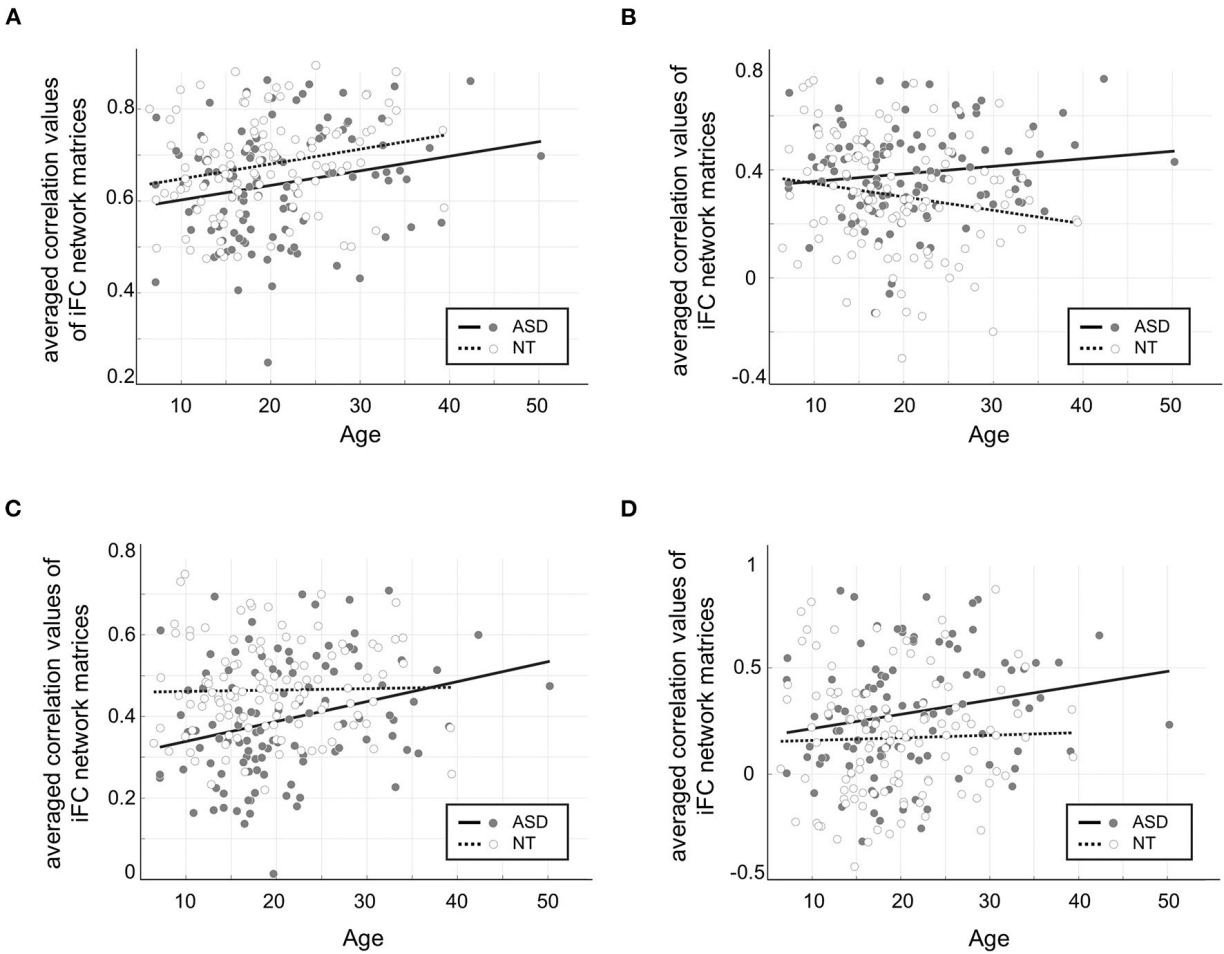
Age by group interaction effects in the connectivities showing significant group differences in previous seed-based analyses. **(A)** Hypoconnectivity among low-level sensory/motor regions in ASD compared to NT was consistently observed across age, but there was no significant interaction effect (*F* = 0.03, *p* = 0.85). **(B)** Hyperconnectivity among low-level cortico-subcortical network connections became increasingly more severe across age in ASD, revealing a significant interaction effect (*F* = 5.27, *p* = 0.02). **(C)** Hypoconnectivity among high-order networks in ASD showed a less severe pattern with age, resulting in a significant interaction effect (*F* = 4.04, *p* < 0.05) **(D)** Hyperconnectivity among high-order networks in ASD showed an increasing age-dependent trend, yet no significant interaction effect (*F* = 1.27, *p* = 0.26).

These effects were tested in the network connections that showed significant group differences in the previous analyses (i.e., within-primary sensory/motor and subcortical networks; within-high-order transmodal systems). Overall, both hypo- and hyperconnectivities in ASD, regardless of which networks are involved, revealed increasing patterns across age (Figure 4). However, these changes in ASD differed depending on whether they are hypo- or hyperconnectivity. The former (i.e., hypoconnectivity within low-level and high-order networks) remained constant or even became less severe with age (Figures 4A,C), while the latter (i.e., hyperconnectivity patterns in both low-level cortico-subcortical networks and higher-order networks) became increasingly more severe across age in ASD (Figures 4B,D).

### Mediation Analysis

**Figure 5:**
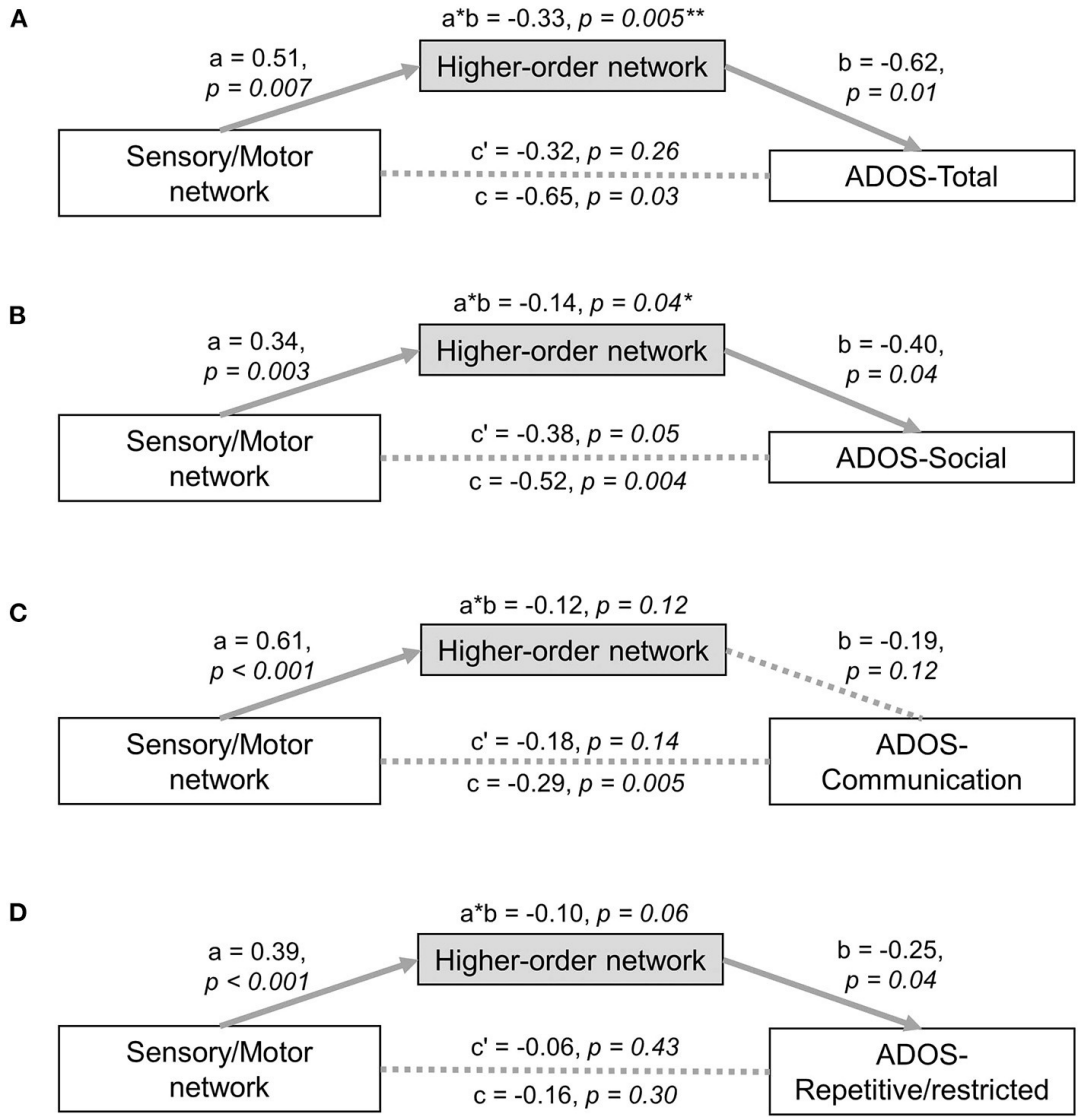
Mediation analysis. Mediation analyses with low-level sensory network as the predictor and high-order network as the mediator were tested with the following ASD symptom severity as a responder: **(A)** ADOS-total score, **(B)** ADOS-social score, **(C)** ADOS-communication score, **(D)** ADOS-repetitive/restricted behavior score. ^*^significant at *p* < 0.05, ^**^survived multiple comparison correction using a false discovery rate procedure, ADOS, Autism Diagnostic Observation Schedule.

We performed mediation analyses assessing the relationship between low-level sensory/motor and high order transmodal systems as well as the behavioural symptoms of ASD in a unified statistical model. The effect between low-level and high-order networks (Figure 5A: effect *a* = 0.51, *p* = 0.007, Figure 5B: effect *a* = 0.34, *p* = 0.003), and that between high-order networks and ADOS total score (effect *b* = −0.62, *p* = 0.01) as well as ADOS social interaction scores (effect *b* = −0.40, *p* = 0.04) were statistically significant after controlling for the effect of low-level networks. Critically, the effect of a mediator (i.e., high-order transmodal iFC) was also significant for the relationship between the low-level iFC and ADOS total (= the sum of social interaction and communication subscores), as well as for social interaction scores (100,000 bootstrapping; ADOS-total: *a* × *b* = −0.33, FDR-corrected *p* = 0.005; ADOS-social: *a* × *b* = −0.14, *p* = 0.04). On the contrary, the direct effect from low-level to symptom severity was not significant for total severity scores (*p* = 0.26) after controlling for the effect of high-order networks, indicating a complete mediation effect. Compared to this, however, regarding social interaction scores, it was marginally significant (*p* = 0.05), indicating a partial mediation effect. This partial mediation effect indicates that there is a remaining association between low-level sensory/motor systems and social interaction symptoms even after accounting for the mediation effects of high-order networks. There were no significant mediation effects for the ADOS communication and repetitive/restricted behavior subscales (Figures 5C,D).

It is worth noting that for mediation analysis, the independent variable (i.e., sensory/motor) is required to have temporal precedence to make any strong claims regarding causality. In the present case, sensory/motor areas can be viewed as having temporal precedence relative to higher order association areas based upon feedforward models of information flow (80–82). However, we would caution against such claims given the associational nature of resting state functional connectivity, as well as the presence of multisynaptic relations and indirect connectivity (i.e., connections between brain areas without physical connectivity). Though, it is worth noting that when we tested an alternative model, in which high-order iFCs were the predictor and low-level sensory/motor iFCs were the mediator, no significant effects were detected. Likewise, when the subcortico-sensory/motor iFCs were the predictor and high-order iFCs were the mediator, the mediation effect was also not significant (Supplementary Figure 2). Moreover, our significant mediation effects were replicated after global mean signal regression, which indicates that this result is not merely a consequence of overall drifting by global mean of iFCs across individuals (Supplementary Figure 3).

## Discussion

Our study focused on connectome-level anomalies in ASD to address system-level substrates underlying co-occurring deficits of sensory/motor anomalies and impaired social cognition. By combining connectopic mapping and targeted seed-based iFC analyses, we observed converging evidence of atypical functional connectivity profiles between the low-level sensory/motor and high-order transmodal cortical networks in autism. Connectopic mapping of sensory/motor regions revealed more excessively segregated connectivity patterns in M1 and V1, which findings were largely replicated in an independent dataset (i.e., ABIDE-II). Seed-based iFC analyses along the cortical hierarchy stream further demonstrated hypo-connectivity within both low-level sensory/motor and high-order cortical systems, while showing marked hyper-connectivity in the subcortico-cortical interaction in ASD. Notably, restricted and repetitive behavior was correlated with regional connectopic gradient changes in the primary motor cortex, while impaired social interaction was mediated by the altered sensory/motor and high-order iFCs in ASD. These findings collectively reinforce the notion of the postulated cascading effects from the low-level sensory/motor areas to the high-order brain functional networks, as well as their potential link to the key symptomatology of ASD, providing critical information to develop potential treatments targeting specific aberrant brain network connectivity (83).

Emerging evidence suggests that the degree of altered sensory sensitivity during early developmental period predicts the impairments of language development and social cognition in later life (84, 85). Supporting this behavioral evidence, a recent neuroimaging study also revealed a disrupted macroscale hierarchy affecting both unimodal and transmodal cortical systems in ASD (62), which are collectively associated with the deficit of social interaction and the restricted/repetitive behaviors. Our mediation analyses further enrich the context of this finding, as we demonstrated that the severity of social impairment (related to higher-order cognitive function) is mediated by the iFC of the transmodal systems, which may be in turn affected by atypical development of early sensory areas in ASD. Notably, the restricted and repetitive behaviors did not show any mediation effect from the transmodal iFCs but rather correlation to the altered motor functional gradients. These distinct brain-behavior associations suggest hierarchy-specific mechanisms depending on the symptom of interests, which is in line with a recent molecular study (86) demonstrating that two dissociable symptom domains (i.e., social vs. restricted/repetitive behaviors) make up the genetic architecture of autism. The neurocognitive accounts related to developmental trajectories of social/communication dysfunction propose that the impairments in low-level, bottom-up processes may lead to diminished experiential learning, which in turn interrupt the development of high-order cognition (87). Our findings are reflective of potential biological substrates underlying this detrimental cascading effect in ASD, corroborating increasingly adapted perception-based mechanistic theories such as erroneous predictive coding (88). In addition, the results of our iFC analyses recapitulated a current perspective on the ASD connectopathies, which are largely summarized into the mosaic patterns of both cortico-cortical hypo-connectivity and subcortical-cortical hyper-connectivity (45, 47, 89–92).

Although, the functional organization of primary regions appeared qualitatively very similar between ASD and neurotypical groups, quantitative statistical analyses found focal changes of gradient in the dorsolateral area of M1. This pattern also revealed significant associations with repetitive/restricted behaviors. Our results join the line of accumulating evidence that repetitive/restricted behaviors are related to deficits in the motor cortex (93–95), suggesting high specificity of our connectopic gradient for brain-symptom mapping. ASD-specific anomalies in the gradient organization were also found in the foveal and peripheral fields of V1, which patterns follow the main direction of the eccentricity observed in the retinotopic mapping. This receptive property is previously known for reflecting the capacity of visual search, or the ability to detect the target amongst distractors (96), which is documented to be superior in ASD along with a detail-oriented perceptual processing style, or enhanced processing of local features at the expense of global information (22, 97). The more extreme values at each end of the gradient indicate an excessively isolated functional organization, which may be potentially induced by abnormally increased local connectivity clustering. Although, further, investigation would be needed to confirm this hypothesis, our finding on “over-segregated V1 connectivity” thus may explain the symptom of the locally-oriented visual processing in ASD (98–101).

Several points should be considered in the interpretation of our findings. First, the mediation analyses were based on the premise that sensory development provides the building blocks for high-order cognitive systems. Unfortunately, objectively quantifying this developmental effect requires longitudinal data, which was not available in this study. However, as our dataset covers a wide range of age, we were instead able to map “cross-sectional” aging-dependent changes of functional connectivity, by performing age by group interaction analyses. We found significant interaction effects in the motor connectivity gradient, which showed more extreme gradient values across age in ASD, indicating a more isolated functional connectome organization, consistent with the results from the group difference analysis. Meanwhile, differential changes in hyper- and hypoconnectivity patterns across age corroborates substantial heterogeneity of functional connectivity network in ASD and thus suggests that the previous inconsistent literature on the hyper vs. hypoconnectivity in ASD should be viewed within the context of dynamic network changes during the life span of this condition.

Secondly, regarding the absence of mediation effects for restricted and repetitive behaviors, it may possibly be related to the limited coverage of a single symptom scale, the ADOS in our study. Indeed, in general, to objectively profile the restricted and repetitive behaviors in individuals with ASD, the symptom scale should include a wide range of distinct behavioral domains encompassing both low-level and higher-order domains, such as motor stereotypic behaviors, daily rituals, and rigid interests in repetitive play (3, 102). While the ADOS is a widely used standardized diagnostic tool for ASD, previous reports have shown that it does not fully capture the multidimensional domain of restricted and repetitive behaviors (103); therefore, much caution would be required when deriving conclusions on the behavioral phenotypes based on a single diagnostic tool.

Finally, the results regarding the upper left motor cortex may seem contradictory because a positive association with clinical symptoms was found despite the ASD group showing “more negative” gradient values. These contrasting results may suggest a complex brain-behavior mechanism, i.e., group differences of functional organization may represent the pathological effects of ASD, whereas, the behavioral association may be reflective of a compensatory process that occurs during development. In fact, repetitive/restricted behaviors and interests have been known to serve as a compensatory strategy against atypical sensory processing, because through ritualistic behaviors, a sense of control and predictability can be established when exposed to sensory-laden situations (104–107). Given this context, in our finding, this compensatory effect at the behavioral level may be represented as an increased gradient value in the brain, a pattern that becomes more similar to the typically developing individuals.

Such behavioral explanability of the “connectopic mapping” technique naturally motivates to think about how we can develop this functional gradient into a robust biomarker in the clinical neuroscience. A recent biomarker study of our group (108) proposed three critical conditions for this purpose, namely reproducibility, reliability and predictive validity. Specifically, first, the biomarker should show high reproducibility across large and multiple independent datasets. Owing to collective data open-sharing efforts in the field such as the ABIDE initiatives, our study was able to demonstrate the reproducibility of some findings. However, the sample size required for reproducibility may vary, depending on the conditions of the biomarker such as the kind of target measures or the number of features per sample (109). A recent study based on more than 10,000 datasets suggested that the brain-behavioral associations become more reproducible with sample sizes of N ≿ 2,000 (110). While this is a clear message emphasizing the necessity of more active community-wise data-sharing efforts, in the meantime, advanced statistical methods such as bootstrapping (111) or dimensionality reduction (112) may reduce model overfitting, increasing reproducibility even in smaller samples. Second, the biomarker should show high test-retest reliability, which represents the consistency of a metric when measuring the same object multiple times (113). This is a desirable trait because the high consistency allows for the increase of an individual identification rate (= individual fingerprint) for biomarkers. Regarding the gradient approach, therefore, we need to optimize the dimensionality reduction algorithm (e.g., Laplacian Eigenmaps) to obtain the best parameters providing the highest reliability of the gradient metrics. Finally, the predictive validity should be systematically tested for a given metric. In ASD research, the clinical variables of interest include the diagnostic label, symptom severity, relevant neuropsychological scores (e.g., Social Communication Questionnaire and the Social Responsiveness Scales) and cognitive performance. The reliability provides only an upper bound of prediction ability (114), and how to further optimize the biomarker beyond the metric quality depends on the domain knowledge and the target biological phenomenon. While there is no established answer for this question, one way to increase predictive validity would be through the testing of the biomarker over the targeted variable (= true positive) with the data as much as available, but also against irrelevant or confounding variables (= true negative) to increase the specificity.

## Conclusion

The present study investigated the role of low-level sensory/motor and high-order transmodal networks in heterogeneous autism symptoms. Our findings demonstrate multiple levels of functional connectivity anomalies along the cortical hierarchy, ranging from atypical gradients in primary sensory/motor areas to diverging patterns of hypo- and hyper-connectivity among the subcortico-cortical and cortico-cortical networks. Moreover, these alterations were shown to be associated with key symptoms in ASD, and replicated in an independent dataset. Collectively, our findings suggest clinical utility of connectopic mapping and targeted iFC analyses, which may be developed into an effective biomarker that takes into consideration the integrative properties of multiple hierarchical functional brain systems in the context of heterogeneous symptom manifestation in ASD.

## Data Availability Statement

The code will be publicly available on the Github page of the corresponding author's lab, and the data can be accessed through the openly-shared ABIDE-data repository (http://fcon_1000.projects.nitrc.org/indi/abide/).

## Ethics Statement

The studies involving human participants were reviewed and approved by Autism Brain Imaging Data Exchange (ABIDE) initiative. Written informed consent to participate in this study was provided by the participants' legal guardian/next of kin.

## Author Contributions

SJH and SP conceptualized the study. SP performed the statistical analyses and wrote the first draft of the manuscript. SJH interpreted the data and edited the manuscript. KVH provided the analysis code and critical comments for study conceptualization. HBC assisted with drafting the manuscript and interpretation of the results. SLV, RAIB, MPM, BCB, and ADM critically revised the entire manuscript for important intellectual content. All authors contributed to the article and approved the submitted version.

## Conflict of Interest

The authors declare that the research was conducted in the absence of any commercial or financial relationships that could be construed as a potential conflict of interest.

## Publisher's Note

All claims expressed in this article are solely those of the authors and do not necessarily represent those of their affiliated organizations, or those of the publisher, the editors and the reviewers. Any product that may be evaluated in this article, or claim that may be made by its manufacturer, is not guaranteed or endorsed by the publisher.
