# Supplementary material for: Atypical Integration of Sensory-to-Transmodal Functional Systems Mediates Symptom Severity in Autism
Source: Front Psychiatry. 2021 Aug 20;12:699813. doi: 10.3389/fpsyt.2021.699813 (PMC8417581; doi:10.3389/fpsyt.2021.699813)
Supplement: Supplementary file 1 [file Data_Sheet_1.pdf]

Supplementary Material

Atypical integration of sensory-to-transmodal functional systems mediates symptom severity in autism

Shinwon Park[1,2], Koen V. Haak[3], Han Byul Cho[1,2], Sofie L. Valk[4,5], Richard A. I. Bethlehem[6,7], Michael P. Milham[8,9], Boris Bernhardt[10], Adriana Di Martino[11], Seok-Jun Hong[1,2,8]*

[1]IBS Center for Neuroscience Imaging Research, Sungkyunkwan University, Suwon, South Korea
[2]Department of Biomedical Engineering, Sungkyunkwan University, Suwon, South Korea
[3]Donders Institute of Brain, Cognition and Behaviour, Radboud University Medical Center, 6500HB Nijmegen, the Netherlands
[4]Otto Hahn Group Cognitive Neurogenetics, Max Planck Institute for Human Cognitive and Brain Sciences, Leipzig, Germany
[5]INM-7, FZ Jülich, Jülich, Germany
[6]Autism Research Centre, Department of Psychiatry, University of Cambridge, Cambridge, UK
[7]Brain Mapping Unit, Department of Psychiatry, University of Cambridge, Cambridge, UK
[8]Center for the Developing Brain, Child Mind Institute, NY, USA
[9]Nathan S. Kline Institute for Psychiatric Research, NY, USA
[10]Montreal Neurological Institute and Hospital, McGill University, Quebec, Canada
[11]Autism Center, Child Mind Institute, NY, USA

Supplementary Table 1. Demographic and clinical characteristics of the ABIDE-II dataset

	**ASD** (n=57)	**NT** (n=59)	Statistical test	
	mean (SD)	mean (SD)	t	p
Age (years)	12.1 (5.89)	16.5 (8.21)	-3.30	0.001
Sex (F/M)	6/51	13/46	2.80[a]	0.09
Mean FD	0.22 (0.21)	0.14 (0.16)	2.28	0.02
ADOS-Total	10.5 (4.17)	NA	NA	NA
ADOS-Repetitive behavior	1.04 (1.43)	NA	NA	NA
ADOS-Communication	3.27 (1.89)	NA	NA	NA
ADOS-Social	7.20 (2.75)	NA	NA	NA

[a]Group difference in sex was evaluated by the χ^2 tests. ASD, autism spectrum disorder; NT, neurotypical control; SD, standard deviation; F, female; M, male; FD, framewise displacement; ADOS, Autism Diagnostic Observation Schedule; NA, not available

Supplementary Table 2. Participants profiles based on ADOS modules

	Number of participants (n)	Age, mean (SD)
ABIDE-I ADOS[1]		
Module 2	1	18.4 (NA)
Module 3	40	14.4 (3.67)
Module 4	59	24.9 (7.26)
Not specified	7	23.7 (9.8)
ABIDE-II ADOS[1]		
Module 2	3	7.84 (4.46)
Module 3	30	9.09 (3.28)
Module 4	24	16.4 (5.97)
Not specified	NA	NA

[1]Scores were derived based on: Lord C, Risi S, Lambrecht L, Cook EH, Leventhal BL, DiLavore PC, Pickles A, Rutter M. The Autism Diagnostic Observation Schedule—Generic: A standard measure of social and communication deficits associated with the spectrum of autism. Journal of autism and developmental disorders. 2000 Jun;30(3):205-23.

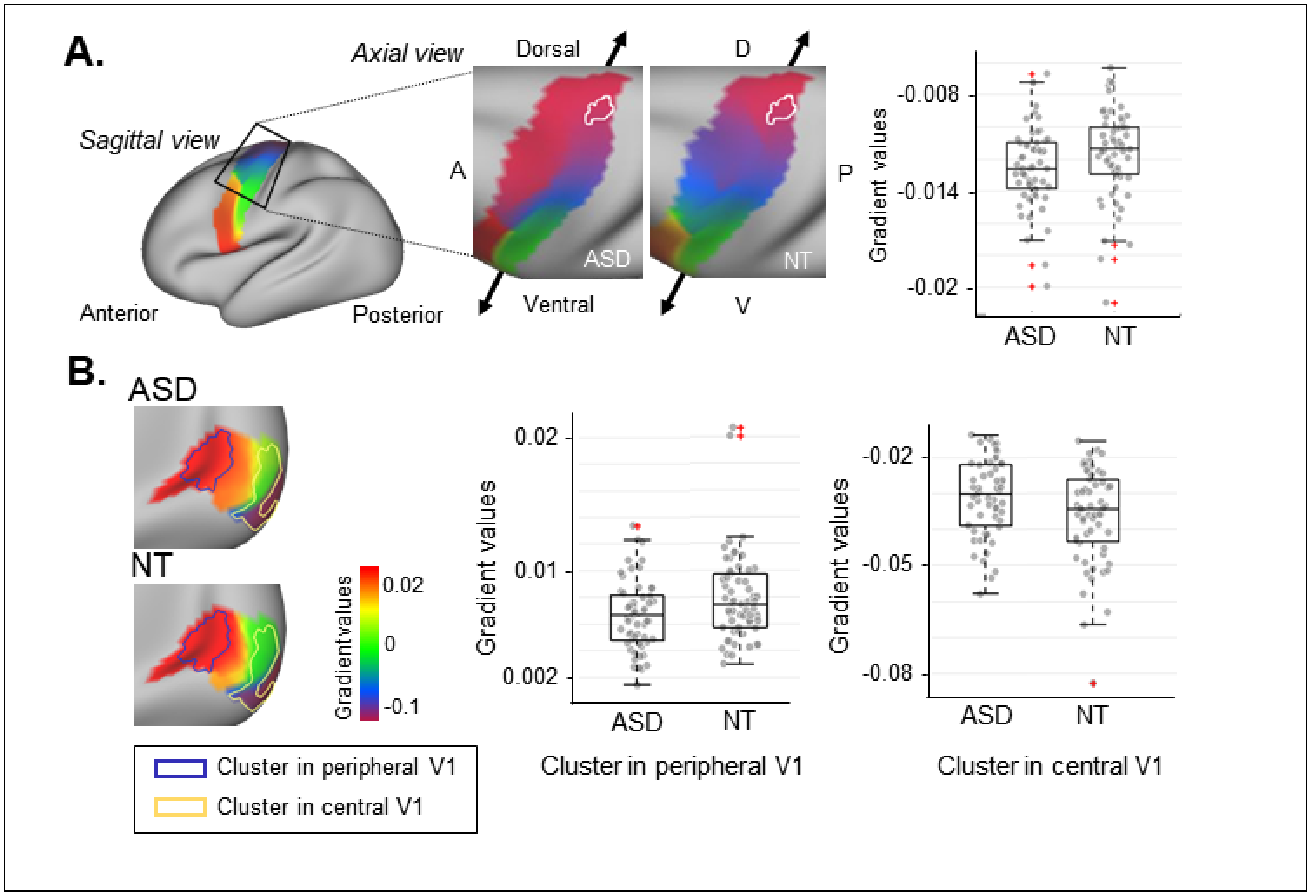

Supplementary figure 1. Replication of functional connectopic gradient analysis using the ABIDE-II dataset. (a) The cluster that showed significant group difference in $M1_{Upper}$ is marked in solid, white outlines. As in the ABIDE-I dataset, more extreme negative gradient scores were found in the ASD group, indicating an abnormally more segregated intrinsic functional connectivity (iFC). (b) The clusters that showed significant group differences in $V1_{Central}$ and $V1_{Peripheral}$ are marked in yellow and blue solid outlines, respectively. ASD, autism spectrum disorder; NT, neurotypical control

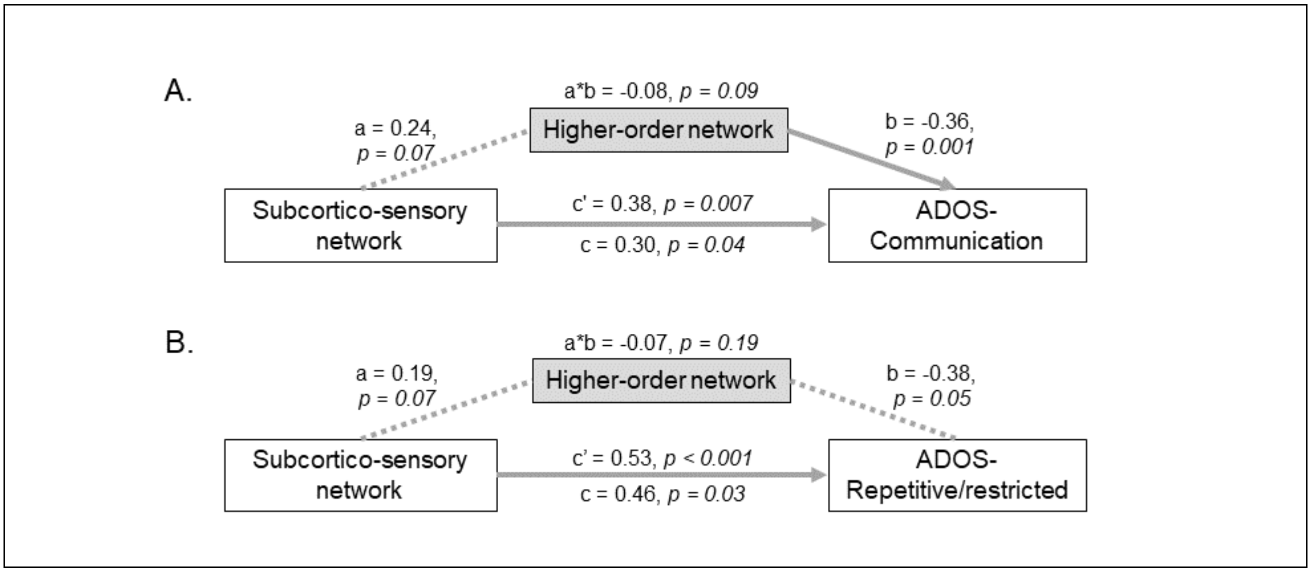

Supplementary figure 2. Mediation models tested with subcortico-sensory/motor as predictor. A series of mediation analyses with subcortico-sensory/motor network as the predictor and high-order network as the mediator were tested. The mediation model was not significant for **A)** ADOS-Communication and **B)** ADOS-Repetitive/restricted behaviors. The predictor did not have any significant iFCs with ADOS-Total and ADOS-Social. ADOS, Autism Diagnostic Observation Schedule, iFC, intrinsic functional connectivity

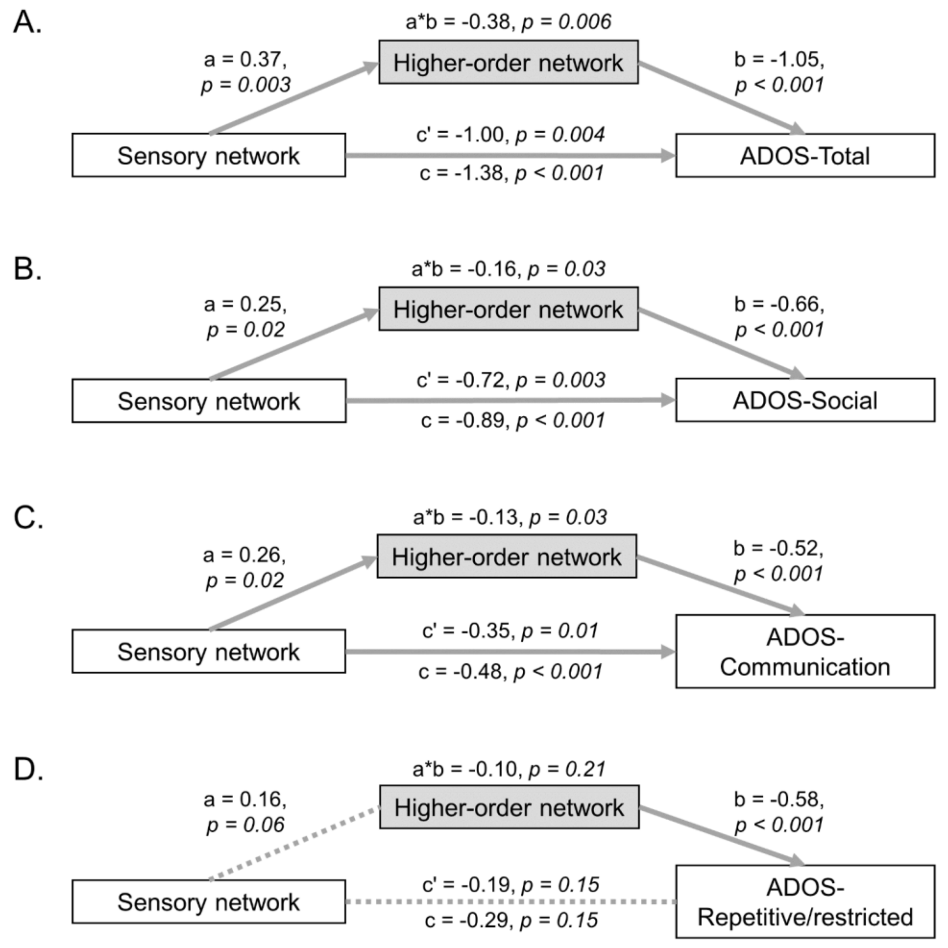

Supplementary figure 3. Mediation models tested after global signal mean regression. A series of mediation analyses with low-level sensory network as the predictor and high-order network as the mediator were tested with ADOS-total and subscores. As in the main analysis, the mediation effect for **A)** ADOS-Total and **B)** ADOS-Social was significant. The mediation model was significant for **C)** ADOS-Communication, only after global signal mean regression. Both mediation models, regardless of global signal mean regression, were not significant for **D)** ADOS-Repetitive/restricted behaviors. ADOS, Autism Diagnostic Observation Schedule
